# Comparative Analysis of the Chloroplast Genomic Information of *Cunninghamia lanceolata* (Lamb.) Hook with Sibling Species from the Genera *Cryptomeria* D. Don, *Taiwania* Hayata, and *Calocedrus* Kurz

**DOI:** 10.3390/ijms17071084

**Published:** 2016-07-07

**Authors:** Weiwei Zheng, Jinhui Chen, Zhaodong Hao, Jisen Shi

**Affiliations:** 1 Collaborative Innovation Center of Sustainable Forestry in Southern China; Key Laboratory of Forestry Genetics and Biotechnology, Ministry of Education, Nanjing Forestry University, Longpan Road 159, Nanjing 210037, China; karrie.zheng@gmail.com (W.Z.); chenjh@njfu.edu.cn (J.C.); haozd1992@163.com (Z.H.); 2 College of Electronics and Information Science, Fujian Jiangxia University, Fuzhou 350108, China

**Keywords:** *Cunninghamia lanceolata* (Lamb.) Hook, coniferous species, chloroplast, phylogeny

## Abstract

Chinese fir (*Cunninghamia lanceolata* (Lamb.) Hook) is an important coniferous tree species for timber production, which accounts for ~40% of log supply from plantations in southern China. Chloroplast genetic engineering is an exciting field to engineer several valuable tree traits. In this study, we revisited the published complete Chinese fir (NC_021437) and four other coniferous species chloroplast genome sequence in Taxodiaceae. Comparison of their chloroplast genomes revealed three unique inversions found in the downstream of the gene clusters and evolutionary divergence were found, although overall the chloroplast genomic structure of the Cupressaceae linage was conserved. We also investigated the phylogenetic position of Chinese fir among conifers by examining gene functions, selection forces, substitution rates, and the full chloroplast genome sequence. Consistent with previous molecular systematics analysis, the results provided a well-supported phylogeny framework for the Cupressaceae that strongly confirms the “basal” position of *Cunninghamia lanceolata*. The structure of the *Cunninghamia lanceolata* chloroplast genome showed a partial lack of one IR copy, rearrangements clearly occurred and slight evolutionary divergence appeared among the cp genome of *C. lanceolata*, *Taiwania cryptomerioides*, *Taiwania flousiana*, *Calocedrus formosana* and *Cryptomeria japonica*. The information from sequence divergence and length variation of genes could be further considered for bioengineering research.

## 1. Introduction

Conifers are the largest and most diverse group of gymnosperms [1,2]. They are distributed widely throughout the world with a total of more than 600 species and 60–65 genera [2]. Most of them have immense economic and ecologic value. *Cunninghamia lanceolata* (Lamb.) Hook (Chinese fir) used to be one of the wide distributed coniferous species across the northern hemisphere during the early Cretaceous to Pliocene periods [3,4,5,6,7,8]. It has remained in the south of China (including Taiwan) [9] and north of Vietnam after the Quaternary glaciation [10]. This species has been cultivated for over 3000 years in China for the ideal traits of disease resistance, rapid growth, wood strength, versatility, high yield in timber production and higher economic value. The present distribution region in China covers the areas from 20 °N to 34 °N in latitude and 100 °E to 120 °E in longitude. There are ~4 million hectares of plantation planted with genetic improved stocks that is intensively managed, which supplies about 40% of the total logs produced by plantations in southern China [11,12]. Although plenty of genetic information is available through the three generations of genetic improvement by conventional strategy [11], an increasing concern is combining traditional breeding with molecular aspects [11,13,14,15,16]. Due to large physical size, slow growth, long generation time, and very large genome, the elucidation of the molecular events on trees, especially on conifers, is very difficult compared with model plants such as *Arabidopsis thaliana* [17]. However, examination of the chloroplast genome is relatively easy [18] and highly informative for many fields such as plant systematics and genetic improvement with chloroplast bioengineering [19,20].

Chloroplasts are the major sites for energy production in plant cells. Typically, chloroplast genomes of higher plants are circular molecules ranging in size from 100 to 200 kb [21] with a pair of inverted repeats (IRs). IRs possess a set of rRNA genes [22], separating the genome into large single-copy (LSC), and small single-copy (SSC) regions. Although the quadripartite structure of chloroplast genome is highly conserved, exceptions have been observed. For example, the chloroplast genomes of some Fabaceae [22,23] and some conifers (including Taxaceae) retain only one segment of the IRs [24,25] and the chloroplast genome of *Euglena gracilis* has three tandem repeats of IR [26]. Chloroplast genomes can thus be categorized into three groups [27]: those that lack one of the IRs, those that possess both IRs and those that contain additional tandem repeats. Presently, plastid genes have been extensively explored in more than 1000 species [28]. Plant chloroplast genomes are highly useful in determining phylogenetic relationships among molecular markers due to their strict inheritance manner without recombination. Based on Kluge’s “total evidence” approach [29], the complete chloroplast genome or several combined sequences have been used for phylogenetic analysis between related species.

The phylogenetic position of *Cunninghamia lanceolata* is a long-standing question in gymnosperm systematics. It was reported that part of the genes of *Cunninghamia lanceolata* were used as a reference sequence in the phylogenetic evolutionary positions for other tree species [30]. The complete chloroplast genome sequence of *Cunninghamia lanceolata* has been announced recently [31]. All of this new progress on chloroplast genome of Chinese fir could provide valuable information for the further research insight into phylogenetic evolutionary biology and chloroplast genomic engineering. In this study, we mainly revisited the published complete Chinese fir (NC_021437) and four other coniferous species chloroplast genome sequence to provide valuable information for Chinese fir evolutionary position demonstrations, and open new avenues for Chinese fir genetic improvement through chloroplast bioengineering.

## 2. Results and Discussion

### 2.1. Re-Characterization of the Cunninghamia lanceolata Chloroplast Genome

**Figure 1:**
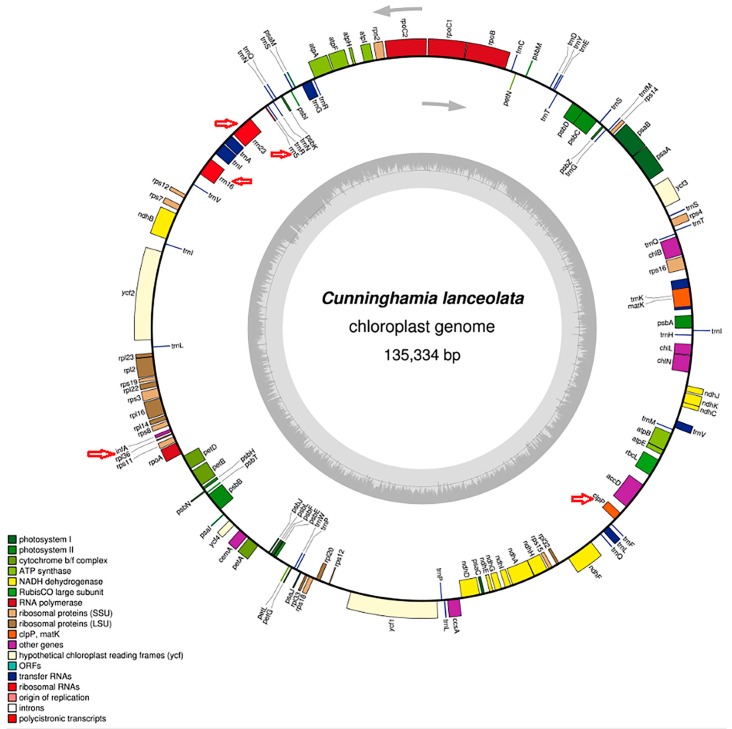
The *Cunninghamia lanceolata* sequences (NC_021437) were re-annotated using DOGMA [33]. The complete genome contains 121 genes. The graphical map of *C. lanceolata* was then generated by OGDRAW [34]. Red arrows indicate new defined genes, including two protein-coding and three rRNA genes.

The genes and their locations are shown in Figure 1. The size of the circular *Cunninghamia lanceolata* chloroplast genome was previously determined to be 135,334 bp [31], which is larger than those of *Pinus thunbergii* (119,707 bp), *Cedrus deodara* (119,299 bp) and *Keteleeria davidiana* (117,720 bp); smaller than the chloroplast genomes of *Cycas revoluta* (162,489 bp) and *Selaginella moellendorffii* (143,780 bp); and approximately the same size as those of *Taiwania cryptomerioides* (132,588 bp) and *Cryptomeria japonica* (131,810 bp). The complete genome contains 121 genes, with two newly defined protein-coding genes and three new rRNA genes.

In Figure 1, we can see that the Chinese fir cp genome contains three rRNA genes (2.5%), 35 tRNA genes (28.9%), four genes encoding DNA-dependent RNA polymerases (3.3%), 21 genes encoding large and small ribosomal subunits (17.4%), 48 genes encoding photosynthesis proteins (39.7%), and nine genes encoding other proteins, in which, proteins with unknown functions (7.4%) are included. Among the 121 genes, 15 contained introns, and *clpP* was identified as a pseudogene. The *C. lanceolata* chloroplast genome has a GC content of 35%, which is similar to that of *Taiwania cryptomerioides* (34%) and of *Cryptomeria japonica* (36%), but lower than that of *Pinus thunbergii* (38%), *Keteleeria davidiana* (38%), *Cycas revoluta* (39%), *Cedrus deodara* (40%) and *Selaginella moellendorffii* (51%). The large IR regions, found in other land plant chloroplast genomes, were not observed in *C. lanceolata*, and therefore the LSC and SSC regions in this genome could not be determined. The function of Large IR was considered to stabilize the cp genome against major structural rearrangements [32]. The large IR regions lost were mostly found in the chloroplast genome of gymnosperms [24] and in the legume family [23]. Heterotachy on the evaluation of gymnosperm phylogeny might be affected by loss of different inverted repeat copies from the chloroplast genomes of Pinaceae and cupressophytes. Because of the highly rearranged and size-variable chloroplast genomes of the conifers II clade (cupressophytes), evolution towards shorter intergenic spacers [25] lead to more gene lose and structural rearrangements in their cp genome [32].

### 2.2. Repeats Analysis

Using Tandem Repeats Finder, 51 repeats were detected in the *Cunninghamia lanceolata* chloroplast genome. Most of these repeats are between 10 and 29 bp in length. Repeats with their length longer than 30 bp are listed in Table 1. The intergenic spacer between *rpl20* and *ycf1* possesses two copies of the longest tandem repeats (185 bp), and the repeat unit at 132 bp in the coding sequence of *ycf2* was the second longest. Most of the repeated sequences are located in protein-coding regions while some are in the intergenic regions (*i.e.*, IGS (*rpl20*, *ycf1*); Table 1). Considering the repeats longer than 30 bp, comparisons were made between the *C. lanceolata* chloroplast genome and those of four other land plants in the Cupressaceae family (*Calocedrus formosana*, *Cryptomeria japonica*, *Taiwania flousiana* and *Taiwania cryptomerioides*). We found that none of the repeat units were shared among these species. In other word, the repeat characteristics in cp genome are unique molecular aspects for those species analyzed.

### 2.3. Chloroplast Genome Rearrangements

**Figure 2:**
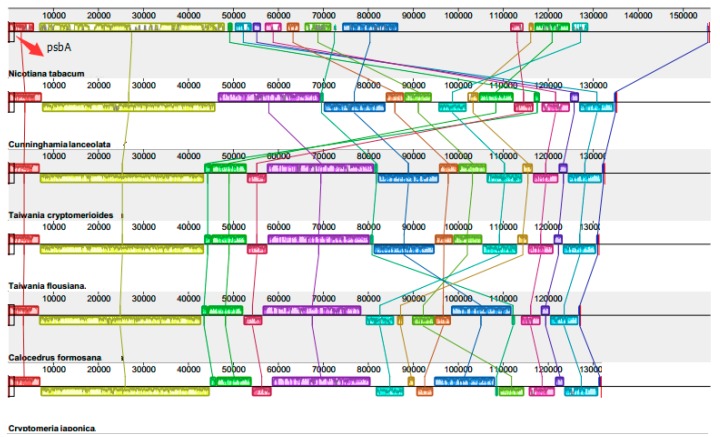
The gene content of five samples in Cupressaceae lineages was visually detected and compared by Mauve [40] with default settings. The colored boxes, which are above and below the middle lines, represent DNA sequences in reverse directions. There were three unique inversions found in the downstream of the gene clusters and evolutionary divergence was shown, although overall the chloroplast genome structure appears to be conserved in the Cupressaceae linage based on the selected plants.

As mentioned in Section 2.1, large IR loss would increase cp genomic rearrangements. The comparison between the *Cunninghamia lanceolata* chloroplast genome and those of four other coniferous species is shown in Figure 2 and Figure S1. *Nicotiana tabacum* is a model plant of angiosperm, and the chloroplast genomic information was reported early [27]. Comparison of cp genome information are made between Chinese fir and *Nicotiana tabacum*, and also among the four species of Taxodiaceae. The results show that *Nicotiana tabacum* appears to be missing two gene regions, which were homologous to the five cupressophytes species. Those two regions are IRs in *Nicotiana tabacum* chloroplast genome. Thus, there is no IR region in those five cupressophytes species. The missing two IRs usually have genes completely or partially missing or losing function compared to those that were in *Nicotiana tabacum*. For example, the *ycf2* was lost with only some homologous sequences and it formed pseudogenes [36,37]. The *ndhB* was lost, which may due to its transferring to the nucleus [36,38,39]. Within the five cupressophytes species, three inversions were found in the downstream of the gene clusters (Figure 2). The first inversion size is ~20 kb and includes the region from *rpl23* to *petA*; the second is 7.5 kb and includes *psbJ* to *rps12*; and the third and smallest inversion is only 2 kb and includes *trnP*, *trnL* and *ccsA* and their flanking sequences. Among the linage, there are some genes completely or partially lost, as well as their functions. It was clear that cp genomic rearrangements occurred, from *C. lanceolata* to *Taiwania cryptomerioides*, *Taiwania flousiana*, *Calocedrus formosana*, and *Cryptomeria japonica*.

### 2.4. Selection Force and Substitution Rate Assessment

**Figure 3:**
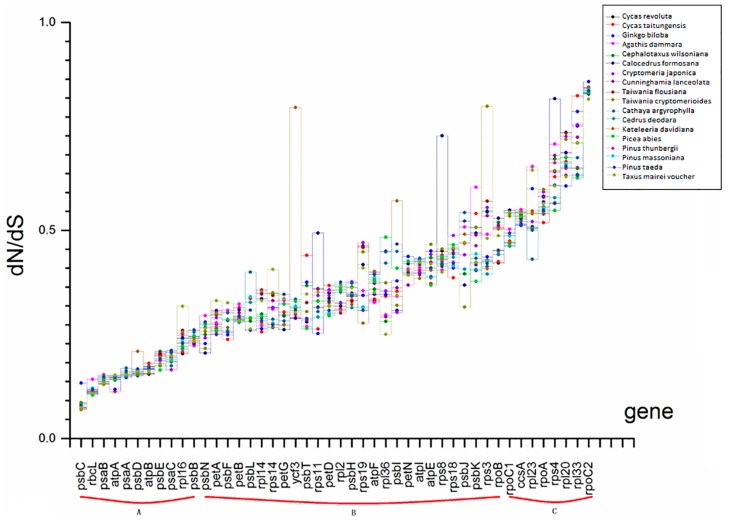
Comparison of the selection forces (dN/dS) of the 46 common protein-coding genes in the 19-species matrix. The matrix consisted of 19 species including *Selaginella moellendorffii* and 18 gymnosperms. A, B and C represent different dN/dS ranges groups according to the description in Section 3.6.

The analyses demonstrated that the selection force and substitution rate were relatively homogeneous among genes, gene groups and lineages. Figure 3 and Figure S2 show the comparisons of the dN/dS ratios (selection force) for the 19-species matrix (*Selaginella moellendorffii* and 18 gymnosperms) and the 45-species matrix (*Selaginella moellendorffii*, 18 gymnosperms and 26 angiosperms), respectively. The dN/dS ratio of *psbC* among lineages was the lowest (≤0.133) in both matrices, indicating purifying selection. In the 19-species matrix, the highest average dN/dS value was for *rpoC2*, and *Ginkgo biloba* had the highest value (0.858) for this gene among all lineages, indicating neutral evolution (Figure 3). Most of the genes examined showed only slight variation among lineages in the 19-species matrix, although there were a few exceptions (*ycf3* and *psbI* in *Keteleeria davidiana*, *rps11* in *Cephalotaxus wilsoniana*, *rps8* and *rsp4* in *Calocedrus formosana*, and *rps3* in *Taiwania cryptomerioides*).

Comparing all of the dN/dS ratios for these genes among the Cupressaceae species, no apparent differences were observed. As shown in Figure S2, the highest average dN/dS ratios for the 45-species matrix were close to 1, indicating neutral evolution. In particular, in *Phyllostachys propinqua*, *Oryza sativa* and *Phyllostachys edulis*, some dN/dS values exceeded 1. The dN/dS values for genes among lineages in the 45-species matrix showed little variation, with a few exceptions (*atpA* in *Typha latifolia*, *petG* in *Eucalyptus globulus*, *rps11* and *rsp8* in *Calocedrus formosana*, *ycf3* in *Keteleeria davidiana* and *rps3* in *Taiwania cryptomerioides*), and no significant variation was seen in the ratios among the Cupressaceae plants.

**Figure 4:**
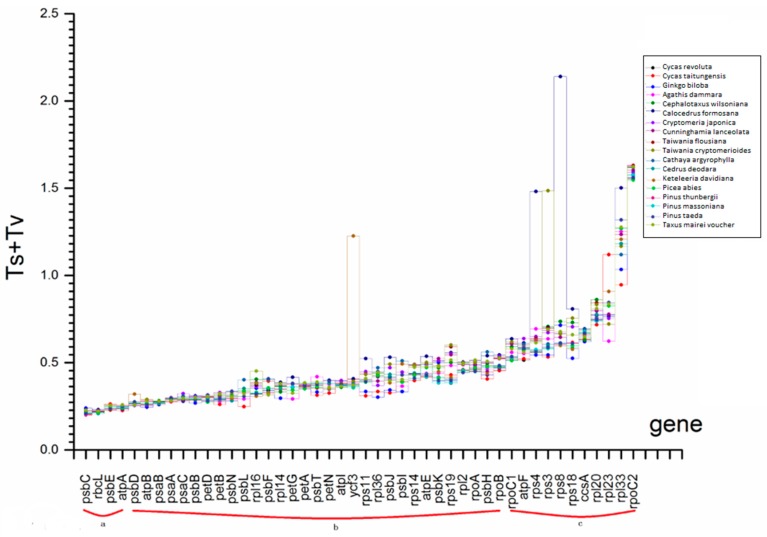
Comparison of the total nucleotide substitution rates (Ts + Tv) of the 46 common protein-coding genes in the 19-species matrix. The matrix consisted of 19 species including *Selaginella moellendorffii* and 18 gymnosperms. a, b and c represent Ts + Tv ranges groups according to the description in Section 3.6.

The total substitution rates among lineages showed a similar pattern to the dN/dS ratios, with some exceptions. The substitution rates for most genes showed little variation among the species in the 19-species matrix, with the exception of *rpl23* and *rpl33* (Figure 4). There was also little variation in Ts + Tv among genes, with a few exceptions (*ycf3* in *Keteleeria davidiana*, *rps4* and *rps8* in *Calocedrus formosana* and *rps3* in *Taiwania cryptomerioides*). The total substitution rates in all Cupressaceae lineages were slightly higher than those of the other lineages. The variation in Ts + Tv among genes showed a similar pattern in the 45-species matrix (Figure S3) as in 19-species matrix.

### 2.5. Phylogenetic Indication Based on Gene Function, Selection Force and Substitution Rate

**Figure 5:**
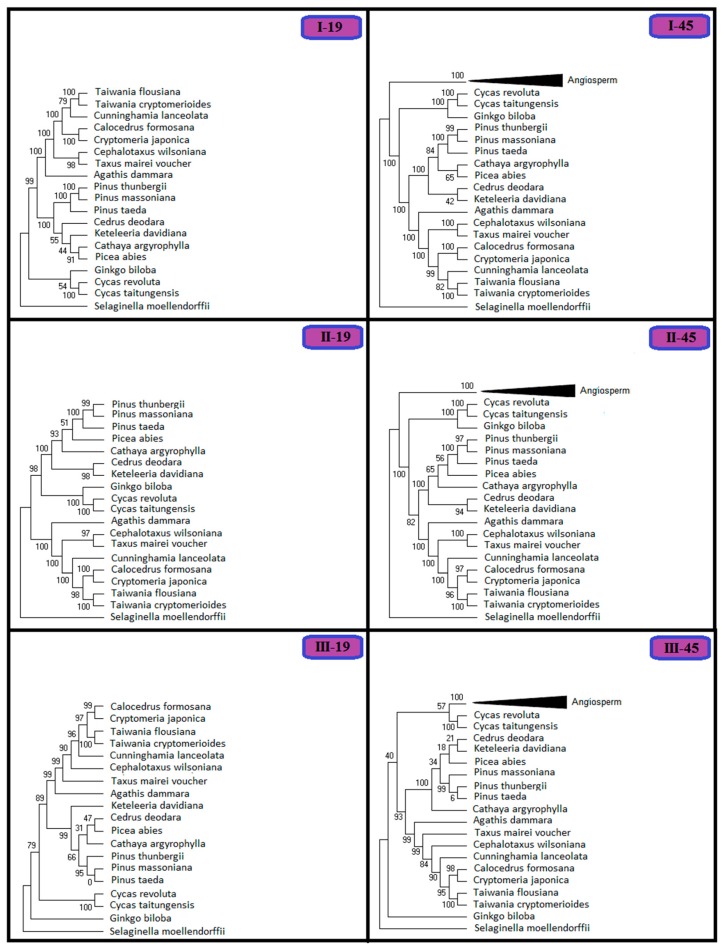
Phylogenetic trees based on the different gene functional groups in the 19-species matrix and the 45-species matrix, respectively. I, II and III represent three main categories of functional genes: (**I**) photosynthetic electron transport and related processes; (**II**) gene expression; and (**III**) other genes.

Phylogenetic analyses was performed both on the data from the 19-species and the 45-species matrices classified according to the three groups for each dataset (I, II and III; Figure 5). Data from the six groups strongly supports that the Cupressaceae lineage is monophyletic, although the topologies of “I-19” and “I-45” demonstrate a sister relationship between *Cunninghamia lanceolata* and *Taiwania flousiana* and between *Cunninghamia lanceolata* and *Taiwania cryptomerioides*, with 79% and 82% bootstrap support, respectively, and the other four phylogenetic trees suggest a sister relationship of *Cunninghamia lanceolata* and the clade containing *Calocedrus formosana*, *Cryptomeria japonica*, *Taiwania flousiana* and *Taiwania cryptomerioides*. Data from these six groups did not clearly resolve the relationships within Pinaceae, as all of the groups contained sub-clades with low bootstrap values (some < 50%).

Phylogenetic analyses were next performed on the data from the 19-species and the 45-species matrices classified according to the selection force range (Figure S4). Results from groups “A-19” and “A-45” support that *Cunninghamia lanceolata* is a sister to *Taiwania flousiana* and to *Taiwania cryptomerioides*, with the same topology as in “I-19” and “II-45”. Data from groups “B-19”, “B-45”, “C-19” and “C-45” strongly support the sister relationship of *Cunninghamia lanceolata* with the *Calocedrus formosana*, *Cryptomeria japonica*, *Taiwania flousiana* and *Taiwania cryptomerioides* clade. The “B-19” and “B-45” trees do not suggest the same monophyletic group of Pinaceae lineages as the other four topologies. Both the “B-19” to “B-45” trees place *Keteleeria davidiana* in the “basal” position among the selected plants instead of *Selaginella moellendorffii*.

In the phylogenetic analyses of the 19-species and the 45-species matrices classified according to the total substitution rates (Figure S5), the topologies were slightly different from the previous analyses based on gene function and selection force. In the “a-19” and “b-45” trees, the relationships between *Cunninghamia lanceolata* and *Taiwania flousiana* and between *Cunninghamia lanceolata* and *Taiwania cryptomerioides* showed low bootstrap values of 68% and 74%, respectively. The topologies for Cupressaceae lineages were consistent and all supported the sister relationship of *Cunninghamia lanceolata* with the *Calocedrus formosana*, *Cryptomeria japonica*, *Taiwania flousiana* and *Taiwania cryptomerioides* clade with high bootstrap values. The “a-45” tree did not clearly resolve the relationships within the selected Cupressaceae lineages, and it shows discordant topology from the analyses based on the substitution rates, with low bootstrap values. The composition of the sub-clade of Pinaceae lineages varied in the six topologies.

In chloroplast genome, heterogeneity of selection force and substitution rate exists in different species/genes [41]. Different selection force and substitution rate have diverse impact on phylogenetic reconstruction although the underlying mechanisms had not yet elucidated completely [42,43,44,45]. Our study (Figure 5, Figures S4 and S5) indicated that three factors, gene functions, selection force and substitution rates, affected phylogenetic reconstruction. Almost all analyses of different data matrices supported sister relationship of *Cunninghamia lanceolata* with the *Calocedrus formosana and Cryptomeria japonica* clade, *Taiwania flousiana* and *Taiwania cryptomerioides* clade, except for the result of using “a-45” data matrix. Thus, three factors’ impacts on phylogenetic reconstruction were further confirmed.

### 2.6. Reconstructing the Phylogenetic Relationships for Gymnosperm Based on Chloroplast Genome

**Figure 6:**
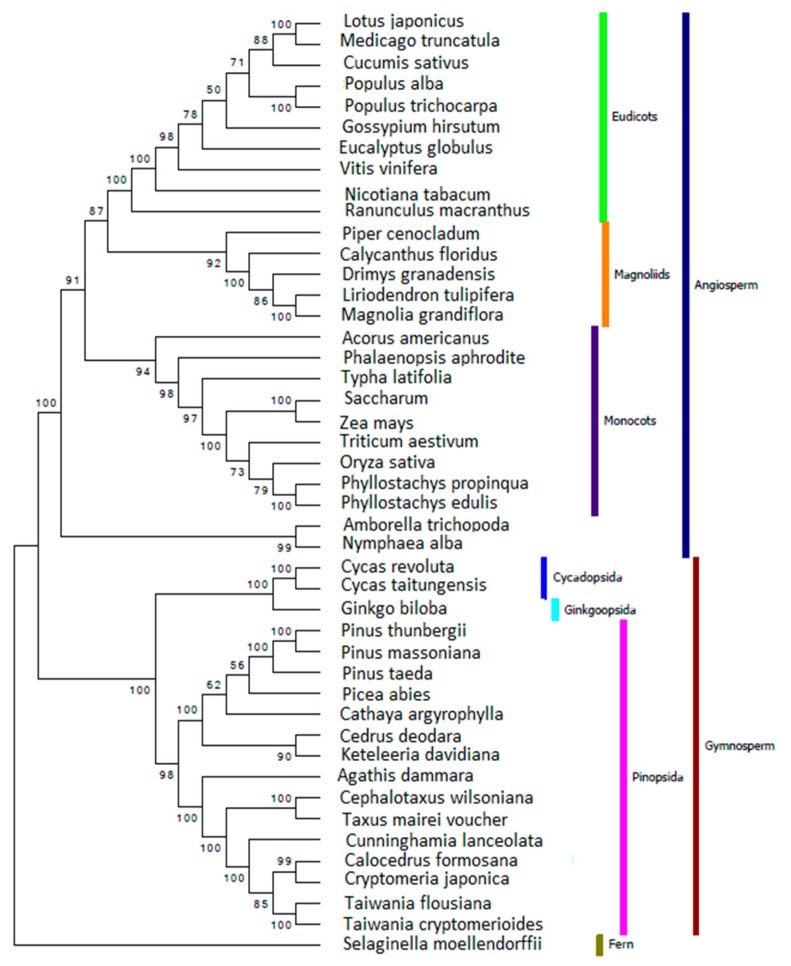
Phylogenetic analyses were performed based on the 65 protein-coding sequences in the 45-species matrix using the maximum likelihood (ML) methods implemented in MEGA5 [46] with the best models [47] calculated using the MEGA5 [46] embedded software “Find DNA/Protein Models” and rapid bootstrapping of 1000 replicates.

The phylogenetic re-analyses based on the 46 common genes in the 19-species matrix, the 46 common genes in the 45-species matrix and the 65 protein-coding genes in the 45-species matrix were shown in Figures S6 and S7 and Figure 6, respectively. All three results suggest the “basal” position of *Cunninghamia lanceolata* among Cupressaceae lineage with slightly different bootstrap values. Figure S6 showed that *Cunninghamia lanceolata* was a sister to *Taiwania cryptomerioides* and *Taiwania flousiana* clade, and to *Calocedrus formosana*, *Cryptomeria japonica* clade with bootstrap value of 100%. In Figure S7 and Figure 6, the value is 85%. All three results 100% support both the relationship between *Taiwania cryptomerioides* and *Taiwania flousiana*, and between *Calocedrus formosana* and *Cryptomeria japonica*.

## 3. Materials and Methods

### 3.1. Genome Sequence Collection

*Cunninghamia lanceolata* plastid genome sequences and available complete chloroplast genome sequences from another 44 plants were obtained from the NCBI organelle genome resource database. With the goals of minimizing missing data and balancing taxon sampling, the 45 samples (Table 2) included *Selaginella moellendorffii* [48] and almost all orders from the gymnosperms (two from Cycadaceae, one from Ginkgoaceae, one from Araucariaceae, one from Cephalotaxaceae, five from Cupressaceae, seven from Pinaceae, and one from Taxaceae) and angiosperms (one from Cucurbitaceae, two from Fabaceae, two from Salicaceae, one from Malvaceae, one from Myrtaceae, one from Ranunculaceae, one from Solanaceae, one from Vitaceae, one from Winteraceae, one from Calycanthaceae, two from Magnoliaceae, one from Piperaceae, one from Acoraceae, one from Orchidaceae, six from Gramineae, one from Typhaceae, one from Amborellaceae, and one from Nymphaeaceae).

### 3.2. Re-Visiting the Chloroplast Genome

The *Cunninghamia lanceolata* sequences were re-annotated with the aid of the Dual Organellar Genome Annotator (DOGMA) [33]. DOGMA is designed to annotate the genes encoding proteins, tRNA and rRNA. Protein-coding genes were re-identified using the BLAST engine against the GenBank sequence database [49], and the conserved protein motifs were manually identified with the aid of the PFAM database [50]. The intron/exon boundaries and the start/stop codons were especially scrutinized during the re-annotation process. All of the identified tRNA genes were re-determined using tRNAscan-SE 1.21 [51] with the default parameters and the source “Mito/Chloroplast”, and the rRNA genes were re-verified using the RNAmmer 1.2 server [52] and refined using the comparative RNA database [53]. The newly located genes (those not identified in the original analysis of the *C. lanceolata* sequence in the NCBI database (NC_021437)) were manually modified by in silico extension using Expressed Sequence Tag and Sequence Read Archive data of *C. lanceolata* from NCBI [54]. The graphical map of *C. lanceolata* was then generated by using the OrganellarGenomeDRAW tool (OGDRAW) [34]. All of the following analyses were conducted on the re-annotated *C. lanceolata* sequence.

In addition, GC content was analyzed for 19 plastid genomes, including *Selaginella moellendorffii* and 18 gymnosperms. Codon usage of *C. lanceolata* was compared with nine other selected plants, including *Selaginella moellendorffii*, six gymnosperms and two angiosperms. Both GC content and codon usage were calculated using MEGA5 [46].

### 3.3. IR Identification and Sequence Repeat Analysis

REPuter [35] was used to locate and count both forward and inverted repeats in the *C. lanceolata* chloroplast genome. The setting was ≥30 bp for repeat size and ≥90% for the identity of repeats (according to hamming distance of 3) [55]. Self-Blast in NCBI BLASTN was used to confirm the remaining IRs visually (dot-plot analysis). Tandem repeats were identified by Tandem Repeats Finder [56] v4.04 with default parameters [57]. Simple sequence repeats (SSRs) were detected by MISA [58] in Perl script, specifying mononucleotide SSRs as more than eight repeat units, di- and trinucleotide SSRs as four repeat units and tetra-, penta- and hexanucleotide SSRs as three repeat units, and allowing a maximum of 100-bp interruption for adjacent microsatellites. All of the repeats found were verified manually, and the redundant results were removed.

### 3.4. Comparative Analysis of Chloroplast Genomes

The annotated *C. lanceolata* chloroplast genome was imported into Mauve [40], as well as four other published complete plastid genomes from species in the Cupressaceae family (*Calocedrus formosana*, *Cryptomeria japonica*, *Taiwania flousiana*, *Taiwania cryptomerioides*) downloaded from the NCBI database. The gene content of these five samples from major genera in Cupressaceae lineages was visually detected and compared by Mauve [40] with default settings.

### 3.5. Selection Force and Substitution Rate Assessment

The 65 protein-coding genes (Table 3) included in the analyses [24] were extracted from the 45 species using the annotation program DOGMA [33]. Of these genes, 19 of them (*psbA*, *psbM*, *psbZ*, *petL*, *psaI*, *psaJ*, *psaM*, *atpH*, *rps2*, *rps7*, *rps12*, *rps15*, *rps16*, *rpl22*, *rpl32*, *cemA*, *clpP*, *matK* and *ycf4*) were missing in at least one species. Two matrices were constructed for the 46 common genes. One matrix consisted of 19 species including *Selaginella moellendorffii* and 18 gymnosperms, and the other consisted of all 45 species. Both matrices were translated into amino acid sequences with Geneious [59], which were aligned by MUSCLE [60] followed by manual inspection and use as a constraint for nucleotide sequence alignment [61]. According to previous reports, the 46 common genes partition into three main categories with eight sub-groups (Table 3): (I) photosynthetic electron transport and related processes; (II) gene expression; and (III) other genes. Synonymous (dS), nonsynonymous (dN) and total nucleotide substitution rates (d = Transitions + Transversions, Ts + Tv) were determined for spermatophytes by comparison to the fern database from Pamilo-Bianchi-Li [62,63] and Kimura’s two-parameter [64] methods in MEGA5 [46] conducted by the previous researches [41,65]. The three parameters were estimated for each of the 46 genes, and the average values for each gene were calculated for later comparison.

### 3.6. Phylogenetic Indication Based on Gene Function, Selection Force and Substitution Rate

With the goal of determining the effects of nucleotide substitution rate, gene function, and selection force on phylogenetic estimation within gymnosperms (especially in Cupressaceae), the phylogenetic analyses were performed according to the following categories (Table 4): with the genes divided into the three functional groups described above, with the genes partitioned into three groups by range of dN/dS values and with the genes divided into three groups according to the range of Ts + Tv values. The genes were sorted into categories by the average dN/dS and Ts + Tv values among lineages. Because most of the 46 genes have dN/dS values between 0.1 to 1.0 and only a few genes have values greater than 1.0. To balance the number of genes in each group, we defined the three selection force groups as group A (dN/dS ≤ 0.25), group B (0.25 < dN/dS ≤ 0.5) and group C (0.5 < dN/dS). The three nucleotide substitution groups were defined as group a (Ts + Tv ≤ 0.25), group b (0.25 < Ts + Tv ≤ 0.5) and group c (0.5 < Ts + Tv). Phylogenetic analyses were performed based on these gene groups for the 19-species and 45-species data matrices using the maximum likelihood (ML) methods implemented in MEGA5 [46] with the best models [47] calculated using the MEGA5 [46] embedded software “Find DNA/Protein Models” and rapid bootstrapping of 1000 replicates.

### 3.7. Reconstructing the Phylogenetic Relationships for Gymnosperms Based on Chloroplast Genome

To determine the phylogenetic position of *C. lanceolata* in gymnosperms (especially in Cupressaceae) and test the possible effects of gene and taxon sampling on this phylogenetic estimation study, we constructed three aligned matrices for phylogenetic analyses. One concatenated matrix consisted of 46 protein-coding plastid genes common among 18 gymnosperms and *Selaginella moellendorffii*. The other two matrices were made up of the 46 and 65 protein-coding plastid genes of 45 plants (including *Selaginella moellendorffii*, 18 gymnosperms and 26 angiosperms). The angiosperms and *Selaginella moellendorffii* served as outgroups to better estimate the topology of the phylogenetic tree. The best-fit nucleotide substitution models [47] for each associated-gene matrix produced by the ML analysis were selected by the MEGA5 [46] embedded function “Find Best DNA/Protein Models”. The ML analyses were performed by MEGA5 with 1000 bootstrap replicates to estimate ML branch support values.

## 4. Conclusions

This study shared gene content, gene order, and intron content of *Cunninghamia lanceolata* by revisiting its chloroplast genome (NC_021437). It also revealed the number of SSRs and tandem repeats. The results provided a well-supported phylogeny framework for the Cupressaceae that strongly confirms the “basal” position of *Cunninghamia lanceolata*. The structure of the *Cunninghamia lanceolata* chloroplast genome showed a partial lack of one IR copy, which is a common feature in gymnosperms chloroplast genomes [31]. The comparison within the Cupressaceae lineage, clearly indicated that rearrangements occurred and slight evolutionary divergence appeared among the cp genomes of *C. lanceolata*, *Taiwania cryptomerioides*; *Taiwania flousiana*, *Calocedrus formosana*, and *Cryptomeria japonica*. Both the sequence divergence and length variation of genes could be further considered for phylogenetic relationship among the lineage [67]. Further attention should be paid to the comparison between the *Cunninghamia lanceolata* chloroplast and nuclear genomes in order to better understand the gene absence/presence and functional transfer in-between [68]. Our study is not only valuable for Chinese fir evolutionary position demonstration, but it would also be beneficial to Chinese fir genetic improvement through chloroplast bioengineering.

## Figures and Tables

**Table 1:** REPuter [35] was used to locate and count both forward and inverted repeats in the *C. lanceolata* chloroplast genome. The minimal repeat size was set to 30 bp and the identity of repeats was set to ≥90%. Fifty-one repeats were detected in the *Cunninghamia lanceolata* chloroplast genome. Most of them are between 10 and 29 bp in length. Repeats longer than 30 bp are listed in the table.

Repeat Number	Size (bp)	Repeat Unit	Location
1	30	AAAAAAGAAAAAATCAACACGAGCAGTAAAA(×2) ^1^	*rpoC2* (CDS ^2^)
2	36	TTGGACGATTTAGAATACGAAACTACATTGGACAAT(×2)	*ycf2* (CDS)
3	132	AAGTATTATTTTCAATGGAAAAAAGCATTCAAAAGATACTATATTGAATTCATAAAAACATTGAATAAGTATTATTTTGAATGGAAAAAAGTATTATTTTGATTCTGTATTAAATTCATAAAAACATTGAAT(×2)	*ycf2* (CDS)
4	66	AAGTATTATTTTGAATGGAAAAAAGTATTAAAAGATTCTGTATTGAATTCATAAAAACATTGAAT(×4)	*ycf2* (CDS)
5	94	TTACGAGCAATAATGAAACAAAACTTGCCAAATACAATGATGACATTATATAATGATACATAGAGATATTGTGTTGCGTTGTTTACAAAACATG(×2)	IGS ^3^ (*rpl20*, *ycf1*)
6	104	CAAAACTTGCCAAATACAATGATGACATTATATAATGATACATAGAGATATTGTGTTGCGTTGTTTACAAAACATGTTACGAGCAATAATGAAACAAAACTTGT(×2)	IGS (*rpl20*, *ycf1*)
7	119	ACAAAACTTGACAAAACTTGCCAAATACAATGATGACATTCTATAATGATAAATAGAGATATTGTGTTGCGTTGTTTAAATGTTACGAGCAATAATGAAACAAAACTTGTCAAAACTG(×2)	IGS (*rpl20*, *ycf1*)
8	185	GGAAAAACAAAAAGAACAAATTGAAAGAATAAGATGCTTAAAATTGACTAATAATATTTTTTTTAATGCAACAAAAATTATTTTAAATACCACTACCACAGGAGGGATATGATCACCACTTTTGCATTGTCTTGGCTACAAAGATGTAGCCCAATAATATTGTTTGGTTTCTATTATGGTTTTTT(×2)	IGS (*rpl20*, *ycf1*), *ycf1* (CDS)
9	30	GAAAAGAAAAGAGAAAAGAACAAGAAGCAT	*ycf1* (CDS)
10	66	ATGAATGAGGCAAAGGATACAAAAATAGACTCCATAACTTCGTCTCAAATGGACTCTTTTTGTAGC(×2)	*ycf1* (CDS)
11	44	TTATTATCTCTTCTAAAATTATTTTGAAAGATCTGATTCAATGG(×2)	*ycf1*, IGS (*ycf1*, tmp)
12	44	CTCTTCTAAAATTATTTTGAAAGATCTGATTCAATGGTTATAAC(×2)	*ycf1*, IGS (*ycf1*, tmp)
13	33	TTTGTTTCAATATTTTCAGAATCTTTGTTTTCC(×3)	*accD* (CDS)

^1^ Parenthetical information refers to repeat numbers. For example, (×2) indicates the number of the repeat unit is 2; ^2^ CDS = coding sequence; ^3^ IGS = intergenic spacer.

**Table 2:** 45 chloroplast genomes selected from *Selaginella moellendorffii* and almost all orders from the gymnosperms and angiosperms in order to minimize missing data and balance taxon sample.

NO.	Taxon	Family	Gneus	Accession Number	NO.	Taxon	Family	Gneus	Accession Number	NO.	Taxon	Family	Gneus	Accession Number
1	*Selaginella moellendorffii*	Selaginellaceae	Selaginella	NC_013086	16	*Pinus thunbergii*	Pinaceae	Pinus	NC_001631	31	*Calycanthus floridus var. glaucus*	Calycanthaceae	Calycanthus	NC_004993
2	*Cycas revoluta*	Cycadaceae	Cycas	NC_020319	17	*Pinus massoniana*	Pinaceae	Pinus	NC_021439	32	*Liriodendron tulipifera*	Magnoliaceae	Liriodendron	NC_008326
3	*Cycas taitungensis*	Cycadaceae	Cycas	NC_009618	18	*Pinus taeda*	Pinaceae	Pinus	NC_021440	33	*Magnolia grandiflora voucher NJ016*	Magnoliaceae	Magnolia	NC_020318
4	*Ginkgo biloba*	Ginkgoaceae	Ginkgo	NC_016986	19	*Taxus mairei voucher*	Taxaceae	Taxus	NC_020321	34	*Piper cenocladum*	Piperaceae	Piper	NC_008457
5	*Agathis dammara*	Araucariaceae	Agathis	NC_023119	20	*Cucumis sativus*	Cucurbitaceae	Cucumis	NC_007144	35	*Acorus americanus*	Acoraceae	Acorus	NC_010093
6	*Cephalotaxus wilsoniana*	Cephalotaxaceae	Cephalotaxus	NC_016063	21	*Lotus japonicus*	Fabaceae	Lotus	NC_002694	36	*Phalaenopsis aphrodite subsp. formosana*	Orchidaceae	Phalaenopsis	NC_007499
7	*Calocedrus formosana*	Cupressaceae	Calocedrus	NC_023121	22	*Medicago truncatula*	Fabaceae	Medicago	NC_003119	37	*Phyllostachys propinqua*	Gramineae	Phyllostachys	NC_016699
8	*Cryptomeria japonica*	Cupressaceae	Cryptomeria	NC_010548	23	*Populus alba*	Salicaceae	Populus	NC_008235	38	*Oryza sativa Japonica Group*	Gramineae	Oryza	NC_001320
9	*Cunninghamia lanceolata*	Cupressaceae	Cunninghamia	NC_021437	24	*Populus trichocarpa*	Salicaceae	Populus	NC_009143	39	*Phyllostachys edulis*	Gramineae	Phyllostachys	NC_015817
10	*Taiwania flousiana*	Cupressaceae	Taiwania	NC_021441	25	*Gossypium hirsutum*	Malvaceae	Gossypium	NC_007944	40	*Saccharum hybrid cultivar NCo 310*	Gramineae	Saccharum	NC_006084
11	*Taiwania cryptomerioides*	Cupressaceae	Taiwania	NC_016065	26	*Eucalyptus globulus* subsp. *globulus*	Myrtaceae	Eucalyptus	NC_008115	41	*Triticum aestivum*	Gramineae	Triticeae	NC_002762
12	*Cathaya argyrophylla*	Pinaceae	Cathaya	NC_014589	27	*Ranunculus macranthus*	Ranunculaceae	Ranunculus	NC_008796	42	*Zea mays*	Gramineae	Zea	NC_001666
13	*Cedrus deodara*	Pinaceae	Cedrus	NC_014575	28	*Nicotiana tabacum*	Solanaceae	Nicotiana	NC_001879	43	*Typha latifolia*	Typhaceae	Typha	NC_013823
14	*Keteleeria davidiana*	Pinaceae	Keteleeria	NC_011930	29	*Vitis vinifera*	Vitaceae	Vitis	NC_007957	44	*Amborella trichopoda*	Amborellaceae	Amborella	NC_005086
15	*Picea abies*	Pinaceae	Picea	NC_021456	30	*Drimys granadensis*	Winteraceae	Drimys	NC_008456	45	*Nymphaea alba*	Nymphaeaceae	Nymphaea	NC_006050

**Table 3:** The 65 protein-coding genes in 45 representative species were extracted from NCBI for construction of the phylogenetic trees [24]. Nucleotides were translated into amino acids using Geneious [59]. Amino acid sequence homologies were aligned by MUSCLE [60]. Aligned genes were concatenated into functional categories [24,66].

Photosynthetic Electron Transport and Related Processes (I)	Subunits of Photosystem I	*psaA*, *psaB*, *psaC*, *psaI*, *psaJ*, *psaM*
	Subunits of Photosystem II	*psbA*, *psbB*, *psbC*, *psbD*, *psbE*, *psbF*, *psbH*, *psbI*, *psbJ*, *psbK*, *psbL*, *psbM*, *psbN*, *psbT*, *psbZ*
	Subunits of Cytochrome	*petA*, *petB*, *petD*, *petG*, *petL*, *petN*
	Subunits of ATP synthase	*atpA*, *atpB*, *atpE*, *atpF*, *atpH*, *atpI*
	Large subunit of Rubisco	*rbcL*
Gene Expression (II)	DNA dependent RNA polymerase	*rpoA*, *rpoB*, *rpoC1*, *rpoC2*
	Small/Large subunits of Ribosome	*rps2*, *rps3*, *rps4*, *rps7*, *rps8*, *rps11*, *rps12*, *rps14*, *rps15*, *rps16*, *rps18*, *rps19*, *rpl2*, *rpl14*, *rpl16*, *rpl20*, *rpl22*, *rpl23*, *rpl32*, *rpl33*, *rpl36*
Other (III)		*ccsA*, *cemA*, *clpP*, *matK*, *ycf3*, *ycf4*

**Table 4:** The genes were sorted into categories by the gene functions, average dN/dS and Ts + Tv values among lineages. The phylogenetic analyses were performed according to these gene groups in order to determining whether the gene function, selection force and nucleotide substitution rate impact phylogenetic estimation [41].

Category	Category ID	Fields
gene function	I	Photosynthetic Electron Transport and Related Processes
	II	Gene Expression
	III	Other
selection force (dN/dS)	A	dN/dS ≤ 0.25
	B	0.25 < dN/dS ≤ 0.5
	C	0.5 < dN/dS
substitution rate (Ts + Tv)	a	Ts + Tv ≤ 0.25
	b	0.25 < Ts + Tv ≤ 0.5
	c	0.5 < Ts + Tv

## Supplementary Materials

Supplementary materials can be found at http://www.mdpi.com/1422-0067/17/7/1084/s1.

## Abbreviations

LSC	large single copy
SSC	small single copy
IR	inverted repeat
ML	maximum likelihood
