# Supplementary material for: Comparative Analysis of the Chloroplast Genomic Information of Cunninghamia lanceolata (Lamb.) Hook with Sibling Species from the Genera Cryptomeria D. Don, Taiwania Hayata, and Calocedrus Kurz
Source: Int J Mol Sci. 2016 Jul 7;17(7):1084. doi: 10.3390/ijms17071084 (PMC4964460; doi:10.3390/ijms17071084)
Supplement: Supplementary file 1 [file ijms-17-01084-s001.pdf]

Supplementary Materials: Comparative Analysis of the Chloroplast Genomic Information of *Cunninghamia lanceolata* (Lamb.) Hook with Sibling Species from the Genera *Cryptomeria* D. Don, *Taiwania* Hayata, and *Calocedrus* Kurz

Weiwei Zheng, Jinhui Chen, Zhaodong Hao and Jisen Shi

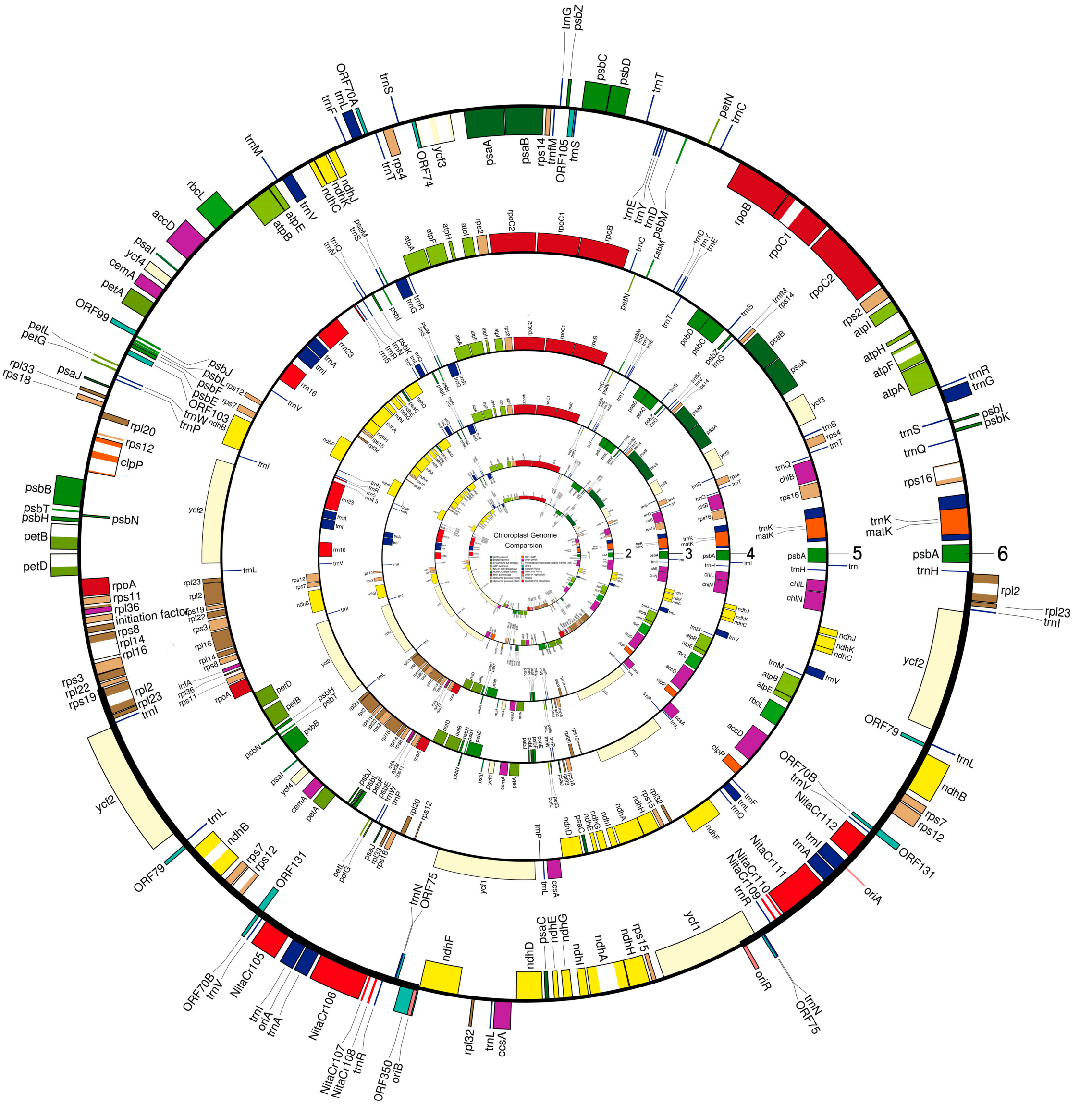

Figure S1. From Outer to inner circles correspond to the cp genome maps: 6-*Nicotiana tabacum*, 5-*Cunninghamia lanceolata*, 4-*Taiwania cryptomerioides*, 3-*Taiwania flousiana*, 2-*Calocedrus formosana* and 1-*Cryptomeria japonica*, respectively. Using the graphical annotation tool DOGMA [33].

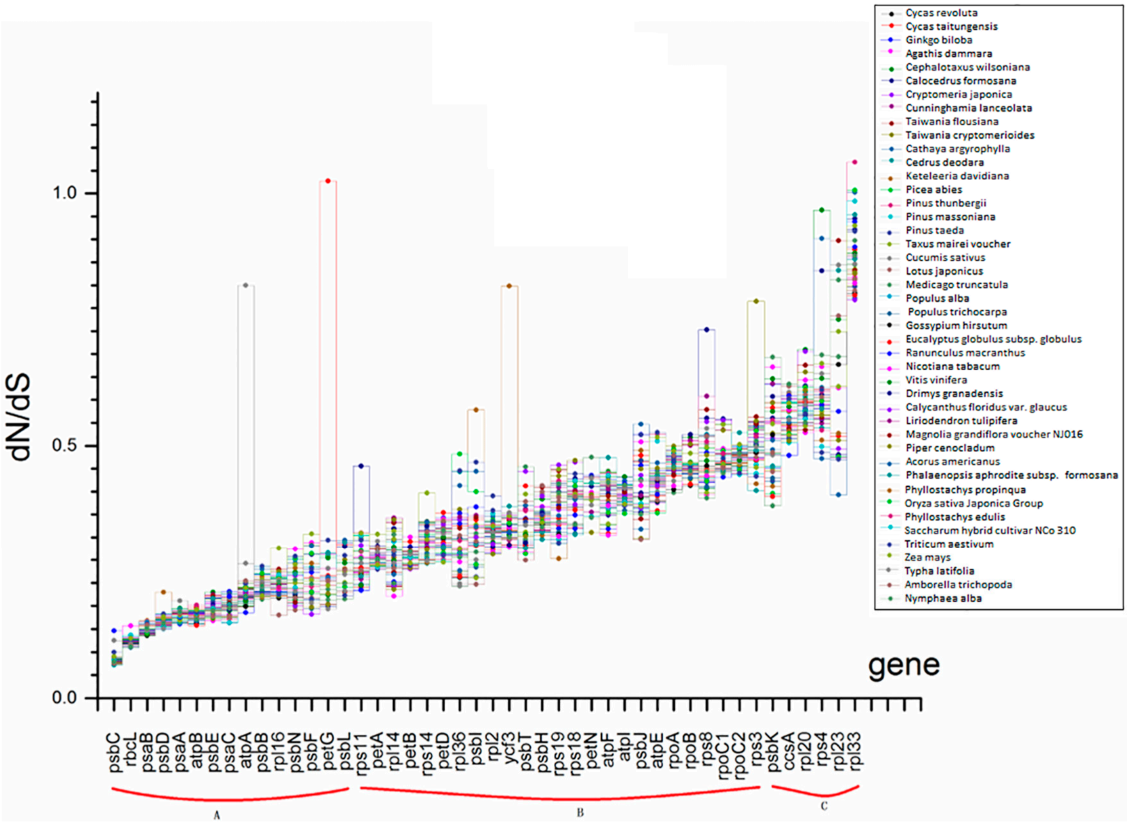

Figure S2. Comparison of the selection forces (dN/dS) of the 46 common protein-coding genes in the 45-species matrix. The matrix consisted of all 45 species listed in Table 2. A, B and C represent different dN/dS ranges groups according to the description in Section 3.6.

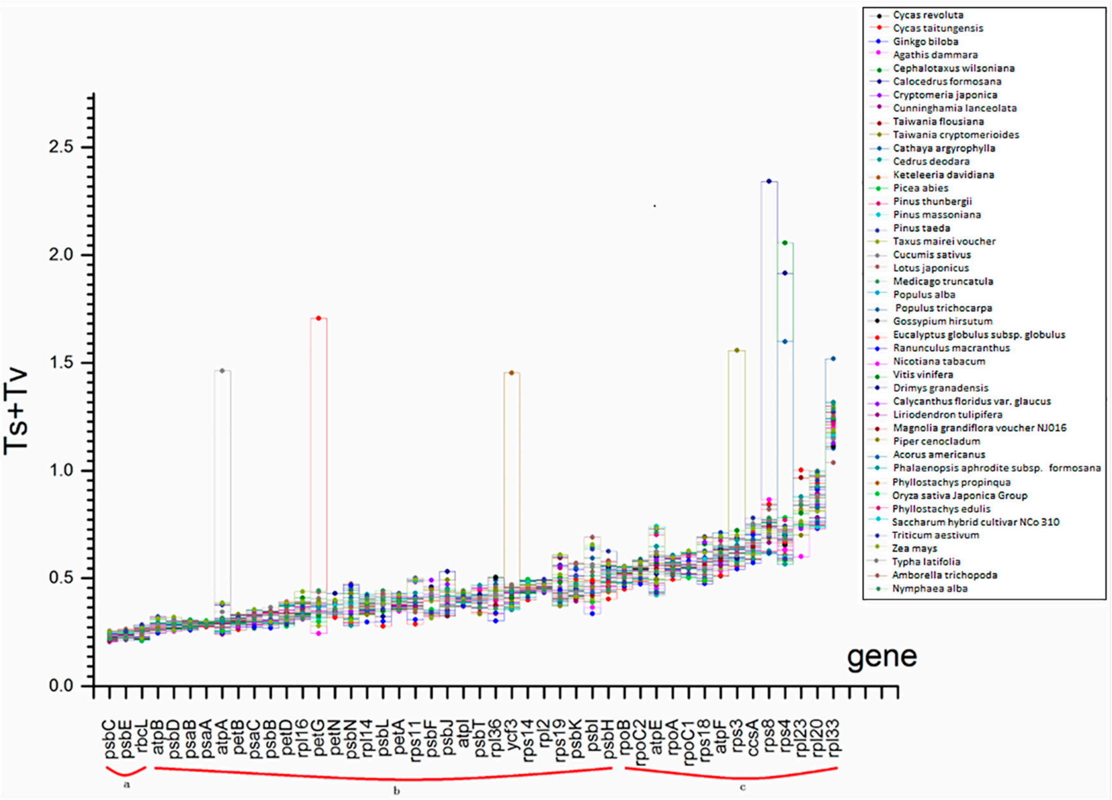

Figure S3. Comparison of the total nucleotide substitution rates (Ts + Tv) of the 46 common protein-coding genes in the 45-species matrix. The matrix consisted of all 45 species listed in Table 2. a, b and c represent Ts + Tv ranges groups according to the description in Section 3.6.

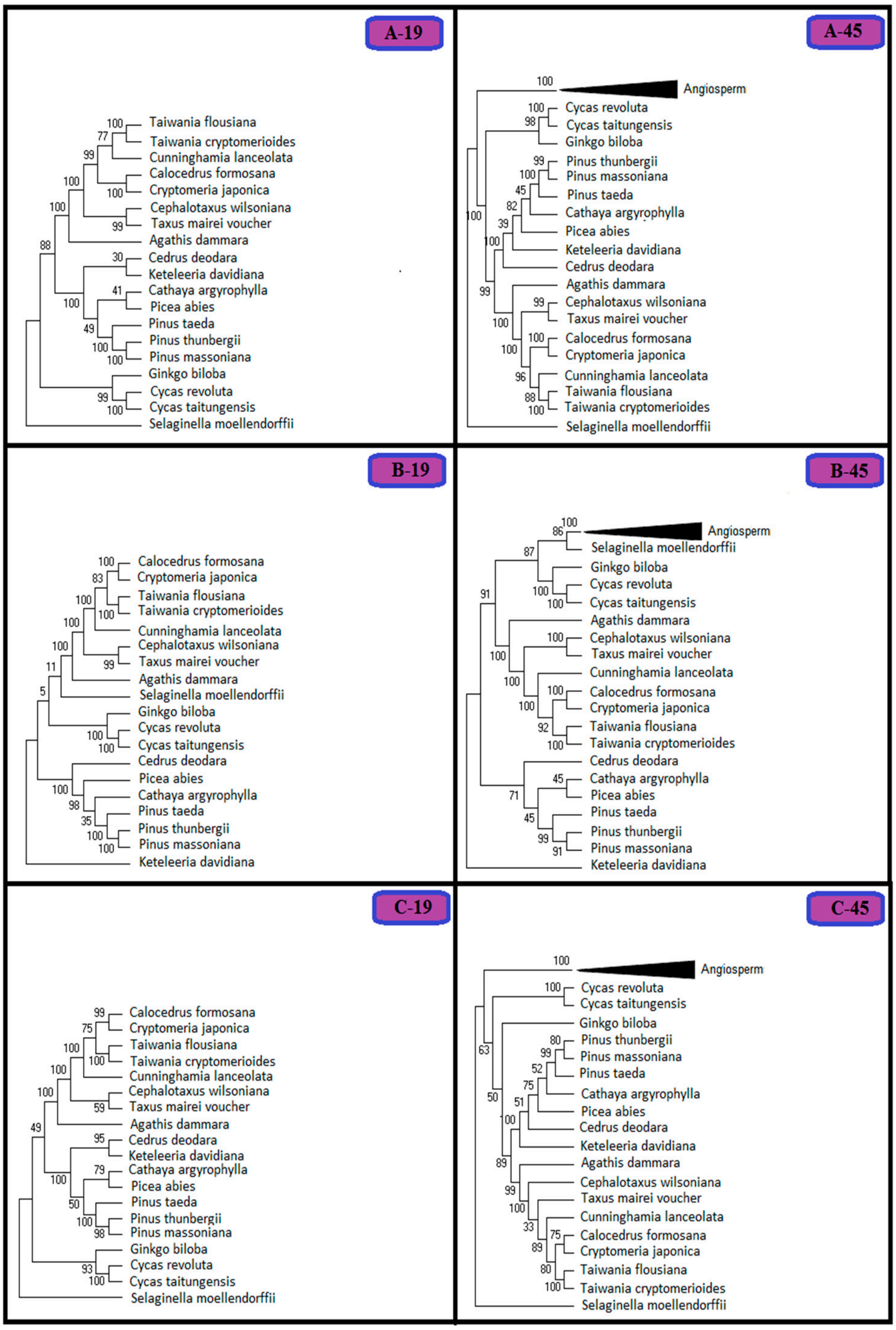

Figure S4. Phylogenetic trees based on the selection force groups in the 19-species matrix and the 45-species matrix respectively. The three selection force groups were defined as group A (dN/dS ≤ 0.25), group B (0.25 < dN/dS ≤ 0.5) and group C (0.5 < dN/dS).

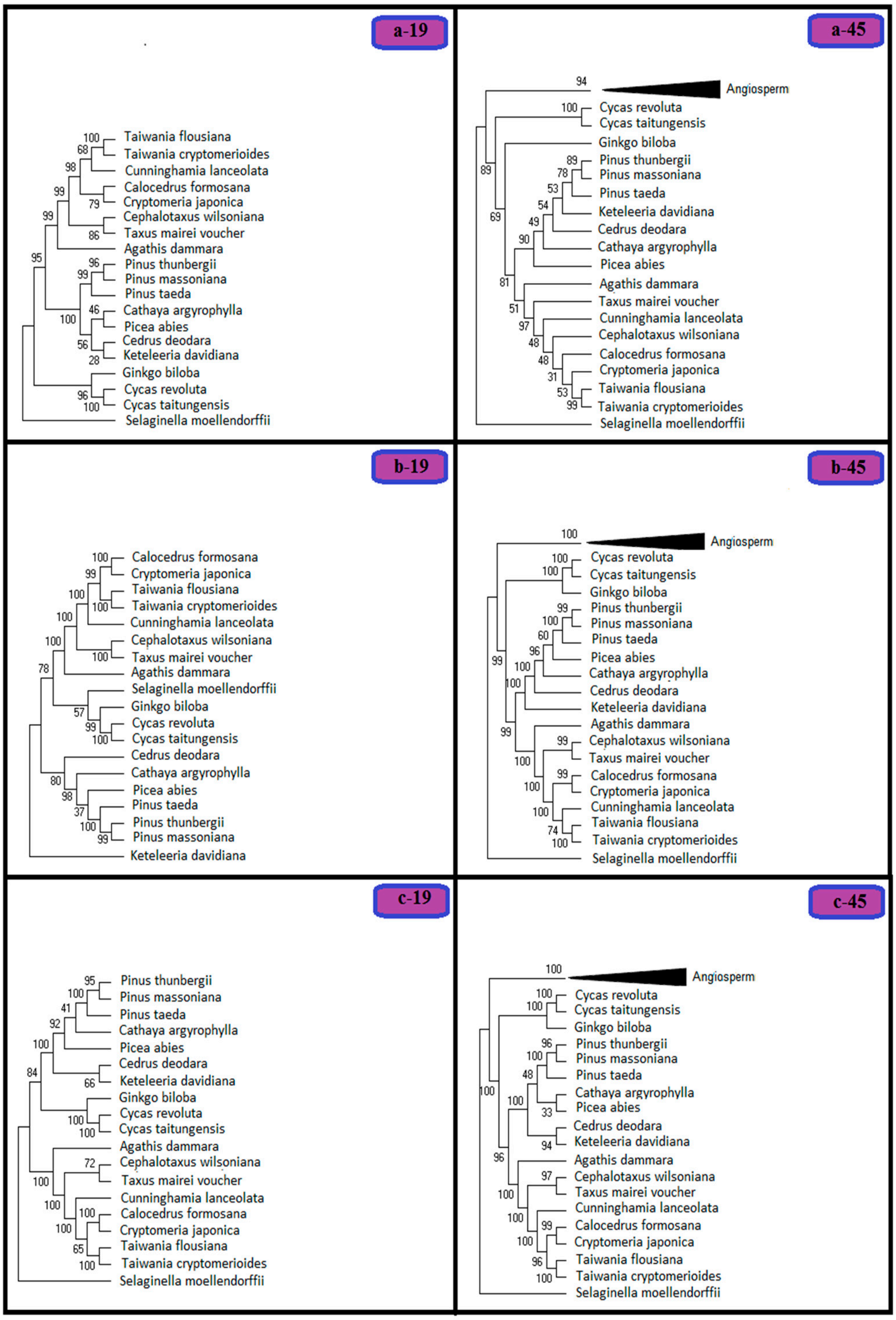

Figure S5. Phylogenetic trees based on the total nucleotide substitution rates in the 19-species matrix and the 45-species matrix respectively. The three nucleotide substitution groups were defined as group a (Ts + Tv ≤ 0.25), group b (0.25 < Ts + Tv ≤ 0.5) and group c (0.5 < Ts + Tv).

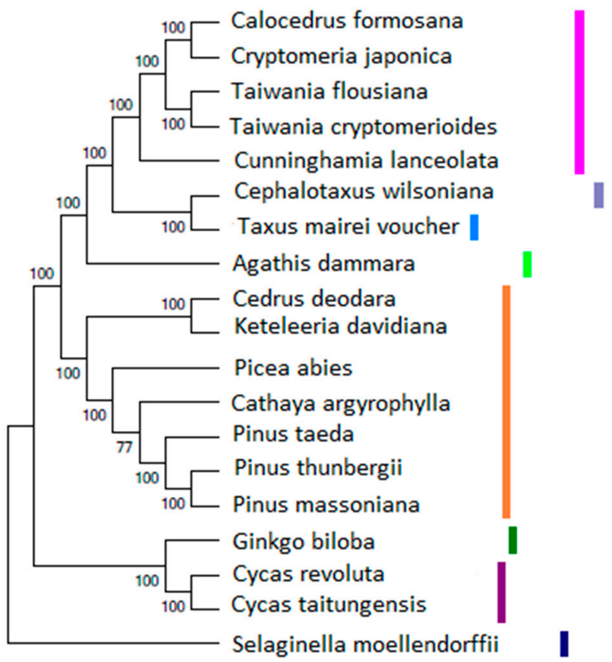

Figure S6. Phylogenetic analyses were performed based on the 46 common protein-coding sequences in the 19-species matrix using the maximum likelihood (ML) methods implemented in MEGA5 [46] with the best models [47] calculated using the MEGA5 [46] embedded software “Find DNA/Protein Models” and rapid bootstrapping of 1000 replicates.

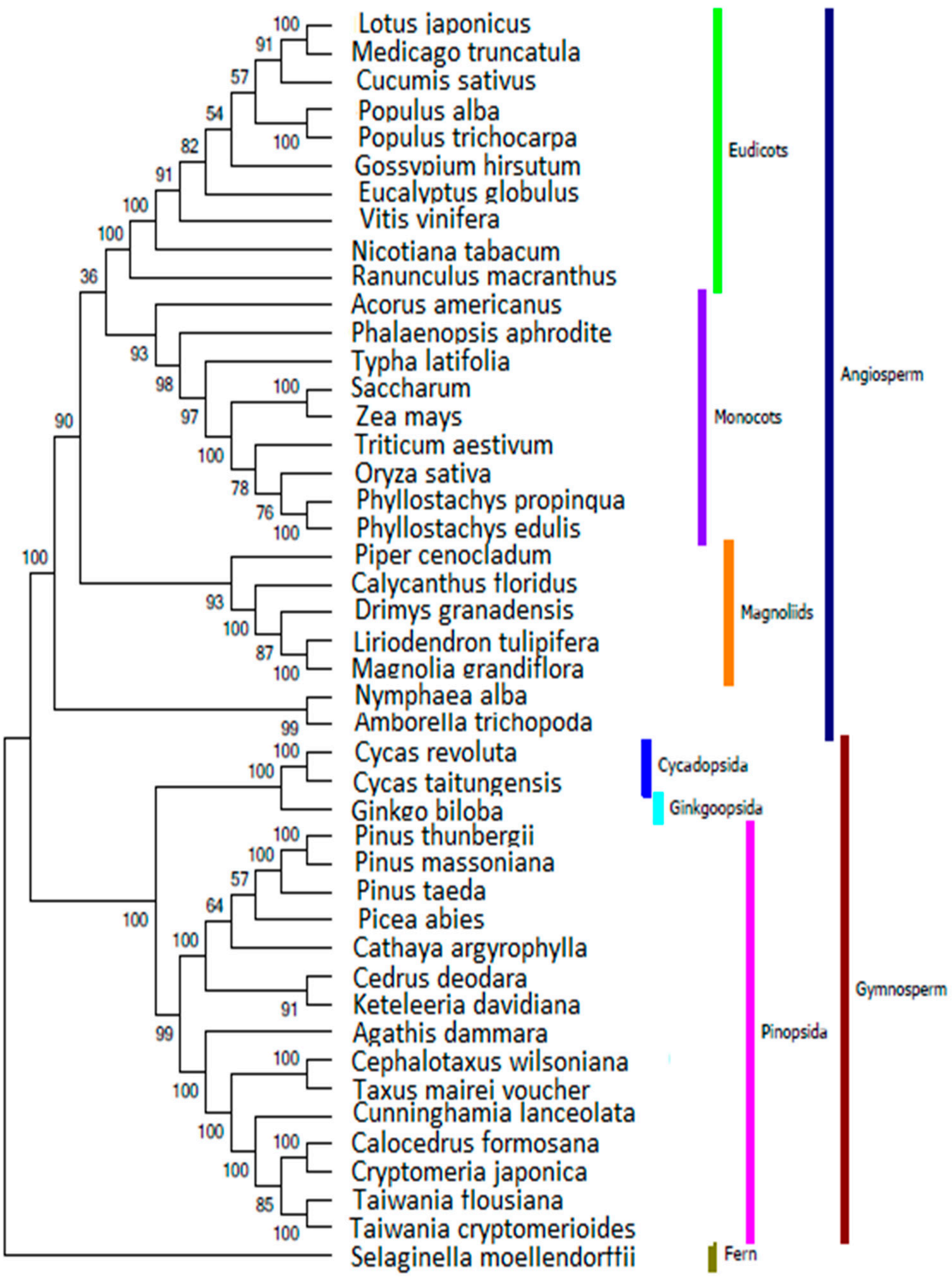

Figure S7. Phylogenetic analyses were performed based on the 46 common protein-coding sequences in the 45-species matrix using the maximum likelihood (ML) methods implemented in MEGA5 [46] with the best models [47] calculated using the MEGA5 [46] embedded software "Find DNA/Protein Models" and rapid bootstrapping of 1000 replicates.
